# Neurocognitive Screening in Patients Following SARS-CoV-2 Infection: Tools for Triage

**DOI:** 10.21203/rs.3.rs-1127420/v1

**Published:** 2022-02-07

**Authors:** Karen Blackmon, Gregory S. Day, Harry Ross Powers, Wendelyn Bosch, Divya Prabhakaran, Dixie Woolston, Otto Pedraza

**Affiliations:** 1. Mayo Clinic, Department of Psychiatry and Psychology, Jacksonville, FL, USA; 2. Mayo Clinic, Department of Neurology, Jacksonville, FL, USA; 3. Mayo Clinic, Division of Infectious Diseases, Jacksonville, FL, USA; 4. Mayo Clinic, Center for Individualized Medicine, Jacksonville, FL, USA; 5. Mayo Clinic, Department of Psychiatry and Psychology, Phoenix, AZ, USA

**Keywords:** COVID-19, long COVID, neuroinfectious disease, neuropsychology, memory, Post Intensive Care Unit Syndrome, myalgic encephalomyelitis

## Abstract

**Background and purpose:**

Cognitive complaints are common in patients recovering from Coronavirus Disease 2019 (COVID-19), yet their etiology is often unclear. We assess factors that contribute to cognitive impairment in ambulatory versus hospitalized patients during the sub-acute stage of recovery.

**Methods:**

Participants were prospectively recruited from a hospital-wide registry. All patients tested positive for SARS-CoV-2 infection using a real-time reverse transcriptase polymerase-chain-reaction assay. Patients ≤ 18 years-of-age and those with a pre-existing major neurocognitive disorder were excluded. Participants completed an extensive neuropsychological questionnaire and a computerized cognitive screen via remote telemedicine platform. Rates of subjective and objective neuropsychological impairment were compared between the ambulatory and hospitalized groups. Factors associated with impairment were explored separately within each group.

**Results:**

A total of 102 patients (76 ambulatory, 26 hospitalized) completed the symptom inventory and neurocognitive tests 24 ± 22 days following laboratory confirmation of SARS-CoV-2 infection. Hospitalized and ambulatory patients self-reported high rates of cognitive impairment (27-40%), without differences between the groups. However, hospitalized patients showed higher rates of objective impairment in visual memory (30% vs. 4%; p=0.001) and psychomotor speed (41% vs. 15%; p=0.008). Objective cognitive test performance was associated with anxiety, depression, fatigue, and pain in the ambulatory but not the hospitalized group.

**Conclusions:**

Focal cognitive deficits are more common in hospitalized than ambulatory patients. Cognitive performance is associated with neuropsychiatric symptoms in ambulatory but not hospitalized patients. Objective neurocognitive measures can provide essential information to inform neurologic triage and should be included as endpoints in clinical trials.

## Introduction

Cognitive complaints are common in patients recovering from Coronavirus Disease 2019 (COVID-19) [1]. In many cases, it may be unclear whether complaints reflect neurologic injury [2] or the combined (and potentially reversible) effects of depression, anxiety, and sleep dysfunction, which are common in recovering patients [3]. Focal neurologic injuries, including ischemic and hemorrhagic strokes, are more common in hospitalized relative to ambulatory patients [2]. Yet, subjectively reported symptoms of brain fog, weakness, fatigue, and myalgia are elevated in ambulatory patients [3], to a higher degree than hospitalized patients in some reports [4]. Different etiological factors likely contribute to neurocognitive complaints in patients with mild versus severe COVID-19, influencing treatment and prognosis. Neuropsychological triage may help disentangle the contributions of focal neurological injuries and non-focal contributions of mood, anxiety, and sleep disorders to neurocognitive deficits in recovering patients.

A prior systematic review concluded that acute illness severity was not predictive of post-acute cognitive outcomes [1]. However, most studies to date have been limited by use of subjective symptom reporting to probe cognitive endpoints [5–8]. Subjective measures are only weakly associated with objective neurocognitive performance in COVID-19 [9]. Among studies that utilized objective measures, most involved hospitalized patients only [9–13], which limits the range of illness severity, or utilized abbreviated screening measures [11 13 14], which limits detection of focal (i.e., domain-specific) cognitive effects. When a more extensive neuropsychological assessment battery was administered to patients approximately 7-8 months after infection, hospitalized patients were more likely to show impairment in attention, executive functioning, verbal fluency, and memory than outpatients [15]; however, the relationship between cognitive performance and neuropsychiatric complaints was not evaluated.

In the current study, we prospectively assessed subjective and objective neuropsychological profiles in a mixed cohort of ambulatory and hospitalized patients. Participants were assessed early in recovery from COVID-19 using a remote, multi-domain, computerized cognitive test platform. We hypothesized that hospitalized patients would show a higher incidence of objective cognitive impairment than ambulatory patients and that elevated depression, anxiety, fatigue, and pain would be associated with lower cognitive performance in ambulatory patients.

## Methods

### Participants

Participants were recruited via email from a hospital-wide registry of patients at Mayo Clinic (Jacksonville, Florida). All patients tested positive for severe acute respiratory syndrome from coronavirus 2 (SARS-CoV-2) infection between June 2020 and March 2021. Infection status was determined by a real-time reverse transcriptase polymerase-chain-reaction (RT-PCR) assay from nasopharynx swab specimens. Participants ≤ 18 years-of-age and those with a history of major neurocognitive disorder were excluded. To participate, patients required access to a desktop or laptop computer for test and survey completion.

### Clinical and Sociodemographic Variables

Patient demographics and health history were extracted from the electronic medical record. The COVID-19 Risk Calculation Score [16] and Covid-19 Disease Severity Score [17] were derived for all participants. Participants were contacted by phone if there was insufficient information in the medical chart to characterize their acute infection symptoms and/or health history.

### Neuropsychological Assessment

Participants completed an extensive neurobehavioral questionnaire (Neuropsych Questionnaire-45) [18] and a computerized cognitive screen (CNS-Vital Signs) [19].

#### Subjective.

The Neuropsych Questionnaire-45 [18] surveys subjective complaints of attention (e.g., difficulty concentrating, easily distracted), memory (e.g., forgetful, misplacing items), anxiety (e.g., feeling nervous, tense, worrying too much), depression (e.g., discouraged about the future, little or no interest in things), fatigue (e.g., low energy, weak), sleep (e.g., hard to fall asleep, disturbed sleep), and pain (e.g., back pain, headache, muscle soreness). Domain scores are summed and classified as minimal (0-74), mild (75-149), or moderate to severe (150-300) problems with neuropsychiatric functioning [18].

#### Objective.

The CNS-Vital Signs core test battery is comprised of 7 subtests: verbal memory, visual memory, finger tapping (motor speed), symbol digit coding, Stroop (selective attention), shifting attention (set-shifting), and continuous performance test (vigilance/sustained attention). From these, 7 domain scores are extracted (Table 1), as described elsewhere [19]. Performance validity indicators are embedded within tests, with cut-off values specified in the CNS-Vital Signs Interpretation Guide [20]. We adjusted domain scores for age based on a normative reference group (mean=100; SD=15), which was collected prior to the COVID-19 pandemic [19]. We classified impairment (< 9^th^ percentile) based on the American Academy of Clinical Neuropsychology consensus conference statement on uniform labeling of performance test scores [21].

### Statistical Analyses

Sample variables were summarized using descriptive statistics. Subjective and objective neuropsychological measures were evaluated for normalcy using the Shapiro-Wilk test following removal of invalid data. Group means, variance, and proportions were derived and compared between the ambulatory and hospitalized groups using t-tests for continuous data or chi-square and Fisher’s exact test for categorical and binomial data, respectively. Nonparametric (Spearman) correlations were used to probe associations between subjective symptom ratings and objective cognitive performance. Level of significance was set at p<0.05, two-sided, and corrected for multiple comparisons using the Benjamini-Hochberg adjusted false discovery rate [22] of 0.014 for the 7 neurocognitive domains that were tested.

### Data Availability

Data not provided in the article and additional information on methods and materials will be shared upon reasonable request from qualified investigators.

## Results

### Sample Characteristics

149 patients consented to study participation. Of these, 102 (76 ambulatory, 26 hospitalized) completed the computerized test battery and symptom questionnaire (68.5%). There were no differences between participants that did and did not complete outcome assessments in age (p=0.15), sex (p=0.72), race (p=0.58), or ethnicity (p=0.47). However, a larger proportion of ambulatory patients did not complete outcome assessments compared with hospitalized patients (p=0.007; Supplementary Table 1). Sociodemographic and clinical characteristics of the cohort that completed outcome assessments are presented in Table 2. There were no differences in the duration of time between laboratory confirmation of COVID-19 infection and assessment between the ambulatory (mean=23.77 days; SD=22.01) and hospitalized (mean=28.76 days; SD=33.02) groups (t=0.14; p=0.89). Twenty-six patients were hospitalized due to respiratory complications/pneumonia (n=23), delirium (n=1), syncope (n=1), and history of chronic respiratory disease without acute pneumonia (n=1). The remainder of the sample remained ambulatory without requirement for supplemental oxygen. Anosmia was reported in 9/26 (35%) hospitalized and 13/76 (17%) ambulatory patients (p=0.095). Age, race and a history of hypertension, coronary artery disease, diabetes, obesity, and smoking were associated with hospitalization status. Five hospitalized patients had documented mental status changes; none had evidence of seizures or stroke.

### Subjective Neuropsychological Complaints

Symptom inventory data were non-normally distributed. Moderate-to-severe problems in attention were reported in 21/73 (29%) ambulatory patients and 9/25 (36%) hospitalized patients (p=0.44). Moderate-to-severe problems in memory were reported in 20/73 (27%) of the ambulatory patients and 10/25 (40%) of the hospitalized patients (p=0.17).

In ambulatory patients, self-reported problems with attention were strongly correlated with the perceived severity of anxiety (rho=0.58; p<0.001), depression (rho=0.65; p<0.001), fatigue (rho=0.69; p<0.001), sleep dysfunction (rho=0.54; p<0.001), and pain (rho=0.39; p=0.001). Self-reported problems with memory were strongly correlated with perceived severity of anxiety (rho=0.59; p<0.001), depression (rho=0.61; p<0.001), fatigue (rho=0.64; p<0.001), sleep dysfunction (rho=0.48; p<0.001), and pain (rho=0.44; p=0.001).

**Figure 1.**
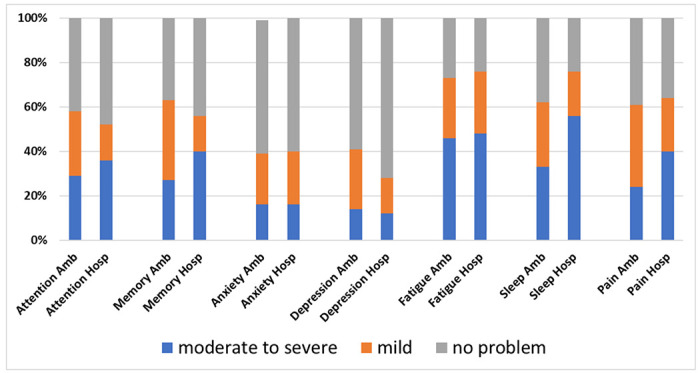
Subjective symptom severity ratings on a self-report inventory. Ambulatory (Amb) patients did not differ in symptom severity ratings from patients who required hospitalization (Hosp) for COVID-19. Amb=ambulatory; Hosp=hospitalized.

Within the hospitalized group, self-reported problems with attention were strongly correlated with perceived severity of anxiety (rho=0.64; p<0.001), fatigue (rho=0.51; p<0.001) and sleep dysfunction (rho=0.73; p<0.001) but not depression (rho=0.38; p=0.07) or pain (rho=0.17; p=0.49). Self-reported problems with memory were strongly correlated with perceived severity of sleep dysfunction (rho=0.58; p=0.003) but not anxiety (rho=0.33; p=0.10), depression (rho=0.37; p=0.06), fatigue (rho=0.35; p=0.09), or pain (rho=0.04; p=0.84; Figure 1).

### Objective Neurocognitive Testing

**Figure 2.**
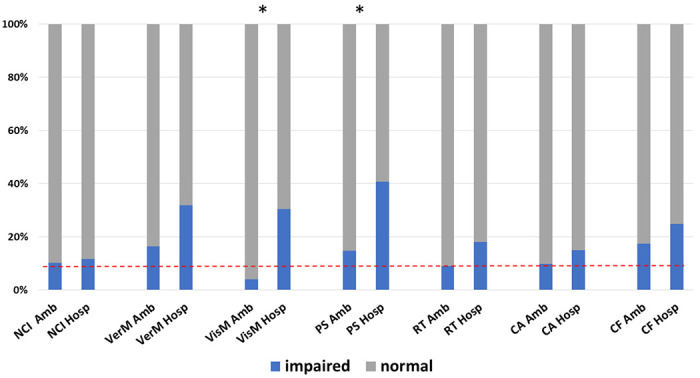
Rates of objective impairment on neurocognitive testing. Patients who were hospitalized for COVID-19 shower higher rates of impairment in visual memory and psychomotor speed compared with patients who remained ambulatory. Impairment was defined by age-adjusted standardized scores <9th percentile (red dashed line = 9%). Asterisk indicates p-value < 0.05.

Neurocognitive performance data were normally distributed. A total of 76/102 (75%) of patients who completed the computerized test battery had sufficiently valid domain scores to derive the overall neurocognitive index (NCI). A total of 8/76 (11%) obtained an NCI score in the impaired range. Rates of overall neurocognitive impairment did not differ between ambulatory [6/59 (10%)] and hospitalized [2/17 (12%)] patients (p=0.85). However, the groups differed in specific cognitive domain performance. The rate of visual memory impairment was higher among hospitalized [7/23 (30%)] than ambulatory [3/73 (4%)] patients (p=0.001). The rate of psychomotor speed impairment was also higher among hospitalized [9/22 (41%)] than ambulatory [11/74 (15%)] patients (p=0.012). Rates of performance impairment for each domain by group are presented in Table 1 and Figure 2. Mean domain scores for each group are presented in Supplementary Table 2. Ambulatory patients performed within the average range on all 7 neurocognitive test domains; among hospitalized patients, mean performance on verbal memory, psychomotor speed, and reaction time measures were in the low average range.

### Predictors of Objective Neurocognitive Performance

We examined the relationship between demographic/clinical factors associated with disease severity (i.e., hospitalization status) and objective performance on the visual memory and psychomotor speed indices to determine whether they may be contributing to the higher impairment rates among the hospitalized group. Within the combined hospitalized and ambulatory groups, there was no relationship between visual memory scores and age (t=0.65, p=0.51), race (χ2=2.27, p=0.53), comorbid hypertension (t=0.75, p=0.46), coronary artery disease (t=−0.70, p=0.48), diabetes (t=−0.07, p=0.94), obesity (t=0.87, p=0.39), or smoking history (t=0.40, p=0.69). There was no relationship between psychomotor speed scores and age (t=0.51, p=0.61), race (χ2=0.35, p=0.95), comorbid hypertension (t=−0.35, p=0.73), coronary artery disease (t=−1.71, p=0.09), or smoking history (t=−0.30, p=0.77). However, reduced psychomotor speed was associated with obesity (t=2.62, p=0.01) and (marginally) with diabetes (t=2.39, p=0.02).

Although education level was marginally associated with hospitalization status, we investigated the relationship between years of education and neurocognitive performance, given that it has served as an important predictor in prior studies [10 12]. Higher education was associated with higher overall NCI (rho=0.29, p=0.014) and psychomotor speed (rho=0.22, p=0.04), but not visual memory (rho=0.16, p=0.14).

**Figure 3.**
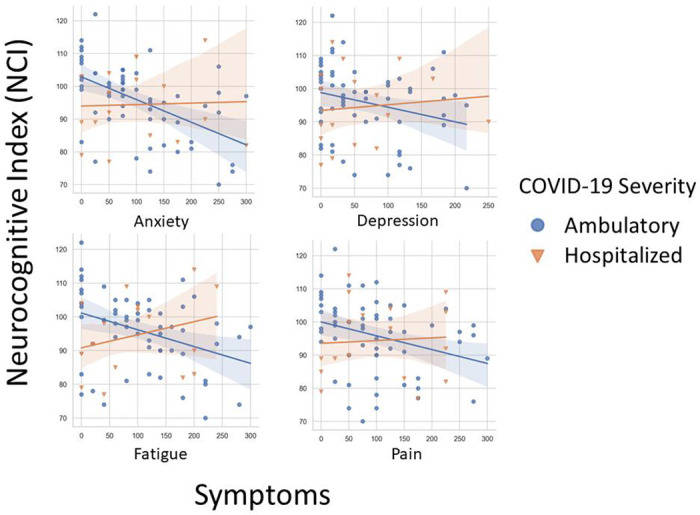
Higher subjective complaints of anxiety, depression, fatigue, and pain are associated with lower objective neurocognitive performance in ambulatory, but not hospitalized, patients.

To determine whether subjective neuropsychological complaints were differentially associated with objective cognitive performance in the ambulatory and hospitalized groups, we analyzed the correlation between NPQ-45 domain scores and the overall NCI (see Figure 3). In ambulatory patients, lower NCI scores were associated with higher subjective complaints of attention (rho=−0.42, P=0.001), memory (rho=−0.57, P<0.001), anxiety (rho=−0.30, p=−.02), depression (rho=−0.28, P=0.03), fatigue (rho=−0.32, P=0.01), and pain (rho=−0.376, p=0.003). In contrast, within the hospitalized group, there were no significant correlations between NCI scores and subjective complaints of attention (rho=0.31, P=0.23), memory (rho=0.34, P=0.70), anxiety (rho=0.22, P=0.41), depression (rho=0.37, P=0.15), fatigue (rho=0.34, P=0.18), or pain (rho=0.15, P=0.57).

## Discussion

Neurocognitive complaints were common in patients recovering from COVID-19 in this series, regardless of disease severity; however, the rate of objective impairment was higher in hospitalized patients. These results emphasize the importance of assessing *both* subjective and objective complaints in determining prevalence of cognitive impairment in recovering patients and research participants. In ambulatory patients, neurocognitive performance was closely linked to depression, anxiety, fatigue, and pain. This was not the case in hospitalized patients. These findings suggest that the drivers of cognitive complaints likely differ in hospitalized versus ambulatory COVID-19 patients. This is important as cause informs treatment. Of particular interest, biopsychosocial factors appear to be a strong driver of cognitive complaints in recovering ambulatory patients. These are treatable factors and interventions targeting anxiety, depression, sleep, and pain may prove to be the most efficient and cost-effective treatment approach to avert disability in patients with mild manifestations of COVID-19 [23 24]. Objective neurocognitive deficits were more common in hospitalized patients—a marker of higher disease severity—with predominant deficits in memory and psychomotor speed. Contributors to focal cognitive deficits in these patients are emerging, representing an important area for future research.

Memory deficits following hospitalization for COVID-19 are increasingly recognized [9 10 14 25]. Previously identified predictors of impairment include hypoxemic respiratory failure [9 10], delirium [10 26], and inflammatory markers [27]. Independent of COVID-19, hypoxia is a known risk factor for long-term deficits in memory [28 29]. Hypoxic/ischemic brain injury is the most common finding on autopsy of patients that died from COVID-19 [30]. Given the presence of respiratory distress in 87% of our hospitalized sample, hypoxic/ischemic injury to hippocampal networks may contribute to the focal memory impairment that we observed. Effects of sustained hypoxemia may be exacerbated by vascular risk factors. Indeed, association between vascular risk factors and objective outcomes were observed in this series, and others [10 31]. Contributions from aberrant acute and chronic inflammation are also expected [11 31 32], with possible unmasking or exacerbation of incipient neurodegenerative disease processes [31], or shared pro-inflammatory risk factors for both Alzheimer’s disease and severe COVID-19 [33]. Longitudinal cognitive tracking is essential to clarify the various ways in which these risk factors interact to compound memory impairment in recovering patients.

In contrast to memory impairment, slower psychomotor speed was associated with comorbid medical conditions, specifically diabetes and obesity. This suggests that a history of diabetes and obesity may contribute to slowing of psychomotor processing speed, independent of COVID-19. A strong association between diabetes and psychomotor speed is already well established [34 35]. Processing speed impairment alone may not be sufficient for justifying extensive neurologic/neuroimaging work-up in patients with comorbid diabetes unless it is associated with other focal neurologic signs.

**Figure 4.**
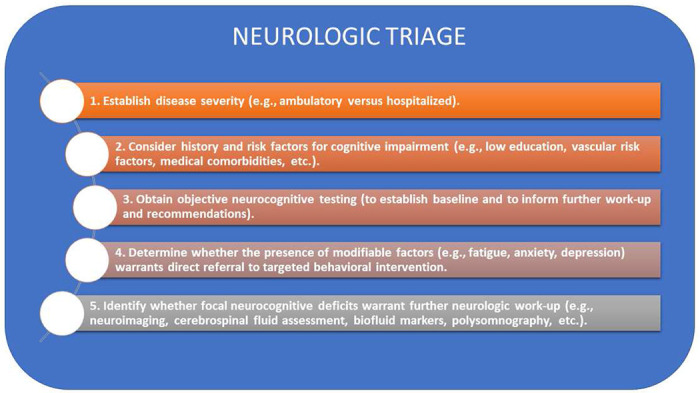
Flow diagram to guide decision-making when patients present with cognitive complaints in the post-acute recovery stage following SARS-CoV-2 infection.

Asymptomatic or mild ambulatory patients performed, on average, within the normal range on objective cognitive measures. This is consistent with a prior study of ambulatory patients who presented to a Neuro-COVID-19 care clinic [3], which found no difference in cognitive test performance between patients who tested positive and negative for SARS-COV-2. Although the SARS-COV-2-positive patients scored lower than a demographic-matched US normative population on measures of attention and working memory, their mean scores were still within the average range [3]. Similar to our findings, they identified a moderate relationship between fatigue and cognitive performance [3]. This suggests that direct referral to behavioral programs designed to address chronic fatigue may offer the most efficient and cost-effective treatment approach for patients complaining of fatigue and ‘brain fog’ [36]. For patients with moderately-to-severely elevated anxiety and depression symptoms, referral for psychiatric evaluation should also be considered. Finally, the detection of focal neurocognitive deficits may warrant further neurological work-up to assess for structural contributors to dysfunction (e.g., neuroimaging to assess for stroke), biofluid markers of inflammation (e.g., cerebrospinal fluid assessment for leukocytosis, or serum measures of neurofilament light [32], or other issues (e.g., polysomnography to assess for occult sleep dysfunction), which may influence long-term prognosis or benefit from specific treatment (Figure 4).

Study limitations include a small sample size of hospitalized patients. A larger proportion of the hospitalized patients in our sample completed outcome assessments compared with ambulatory patients. This suggests that remote computerized testing did not present a disproportionate access barrier for patients with more severe illness. Instances of delirium, seizures, and stroke were limited, precluding direct consideration of the contributions of these events to post-COVID19 subjective complaints and objective impairment. We relied on a 45-minute computerized test battery, which eliminates exposure risk and is accessible to patients from remote locations; however, it requires a home desktop computer and computer literacy. Although this may have biased the sample towards a more socioeconomically advantaged and younger population, there were no differences in age, race, or ethnicity between those who did and did not complete the computerized outcome assessments. This suggests that if patients can electronically sign consent, computerized testing does not present an additional barrier. Given the cross-sectional nature of our study, we are unable to comment on the natural history and long-term risk of COVID-19 cognitive impairment. It will be essential to track cognitive progression at future time points to determine the rate and predictors of cognitive normalization versus decline.

Our study highlights that objective neurocognitive screening procedures should be performed if neurologic manifestations of COVID-19 are suspected based on subjective complaints. The telemedicine-enabled computerized test platform that we utilized appears to be sensitive to disease severity and focal effects (i.e., visual memory impairment) and falls within the scope of level 2 harmonization approach as specified by the NeuroCOVID Neuropsychology Taskforce [37]. Objective neurocognitive testing can inform clinical decision points regarding the need for further imaging or neurologic work-up. In many patients with mild cases of infection and normal performance on objective cognitive measures, it may be appropriate to directly refer them to a multi-disciplinary behavioral rehabilitation program to target mood, anxiety, sleep, and pain symptoms. Finally, our findings highlight the importance of including objective neurocognitive performance measures as endpoints in clinical trials investigating the short- and long-term cognitive consequences of COVID-19.

## Supplementary Material

Supplement 1

## Figures and Tables

**Table 1.** Neurocognitive Test Domains.

Domain Score	Description
Neurocognitive Index (NCI)	Composite of the six neurocognitive domains described below.
Verbal Memory (VerM)	15 target words presented visually, one at a time every 2 seconds, followed immediately by yes/no recognition testing of 15 targets and 15 foils. 15 targets and 15 new foils are presented again after a distraction-filled delay. Correct hits and correct misses are totaled across the immediate and delayed trials.
Visual Memory (VisM)	15 target geometric figures presented visually, one at a time every 2 seconds, followed immediately by yes/no recognition testing of 15 targets and 15 foils. 15 targets and 15 new foils are presented again after a distraction-filled delay. Correct hits and correct misses are totaled across the immediate and delayed trials.
Psychomotor Speed (PS)	Total of right and left taps (in 10 s) from the finger tapping test and total correct responses (in 120 s) from the symbol digit coding test.
Reaction Time (RT)	Average of the two complex reaction time scores from the Stroop test (from the congruent and incongruent trials).
Complex Attention (CA)	Sum of errors from the continuous performance test, shifting attention test, and the Stroop test.
Cognitive Flexibility (CF)	Number of correct responses on the shifting attention test minus the number of errors on the shifting attention test and Stroop test.

**Table 2.** Demographic and clinical characteristics of the cohort

	Overall (N=102)	Ambulatory (N=76)	Hospitalized (N= 26)	*p-value*
**Age**, mean (SD)	52.21 (14.83)	50.41 (14.52)	57.46 (14.72)	0.04
**Education in years**, mean (SD)	15.78 (2.45	16.06 (2.37)	14.96 (2.53)	0.06
**Sex,** n (%)				0.49
Males	44 (43%)	31 (41%)	13 (50%)	
Females	58 (57%)	45 (59%)	13 (50%)	
**Race,** n (%)				0.03
American Indian	0 (0 %)	0 (0%)	0 (0%)	
Asian	5 (5%)	2 (3%)	3 (12%)	
Native Hawaiian	0 (0%)	0 (0%)	0 (0%)	
Black	9 (9%)	4 (5%)	5 (19%)	
White	87 (85%)	69 (91%)	18 (69%)	
More than one race	1 (1%)	1 (1%)	0 (0%)	
**Ethnicity,** n (%)				0.20
Hispanic	8 (8%)	4 (5%)	4 (15%)	
Non-Hispanic	94 (92%)	72 (95%)	22 (85%)	
**The Covid-19 Risk Calculation Score**, n (%)				<0.044
0-2 Points: Low Risk	69 (68%)	56 (74%)	13 (50%)	
3-5 Points: Medium Risk	30 (29%)	19 (25%)	11 (42%)	
>=6 Points: High Risk	3 (3%)	1 (1.3%)	2 (8%)	
**COVID-19 Severity,** n (%)				<0.001
Asymptomatic	11 (11%)	11 (15%)	0 (0%)	
Mild – No Hypoxia	67 (65%)	65 (85%)	2 (8%)	
Moderate - Pneumonia	4 (4%)	0 (0%)	4 (15%)	
Severe - Pneumonia	17 (17%)	0 (0%)	17 (65%)	
Critical - ARDS	3 (3%)	0 (0%)	3 (12%)	
Critical - Sepsis	0 (0%)	0 (0%)	0 (0%)	
**Comorbid Hypertension,** n (%)				0.002
no	66 (67%)	55 (75%)	11 (42%)	
yes	33 (33%)	18 (25%)	15 (58%)	
**Comorbid Coronary Artery Disease,** n (%)				0.015
no	86 (87%)	67 (92%)	19 (73%)	
yes	13 (13%)	6 (8%)	7 (27%)	
**Comorbid Diabetes,** n (%)				0.011
no	89 (90%)	69 (95%)	20 (77%)	
yes	10 (10%)	4 (5%)	6 (23%)	
**Comorbid Obesity,** n (%)				0.046
no	87 (88%)	67 (92%)	20 (77%)	
yes	12 (12%)	6 (8%)	6 (23%)	
**Comorbid Chronic Neurologic Disease,** n (%)				0.953
no	95 (96%)	70 (96%)	25 (96%)	
yes	4 (4%)	3 (4%)	1 (4%)	
**Comorbid Chronic Psychiatric Disease,** n (%)				0.309
no	73 (73%)	56 (76%)	17 (65%)	
yes	27 (27%)	18 (24%)	9 (35%)	
**Smoking History,** n (%)				0.040
Never Smoked	72 (71%)	57 (75%)	15 (58%)	
Past or Current Smoker	26 (25%)	15 (20%)	11 (42%)	
Unknown/Refused	4 (4%)	4 (5%)	0 (0%)	

**Table 3.** Prevalence of cognitive impairment during recovery from COVID-19

	Impaired (< 9^th^ percentile), n (%)			Odds ratio (95% CI)
	Total (N=102)	Ambulatory (N=76)	Hospitalized (N= 26)	Hospitalized vs Ambulatory
Neurocognitive Index	9 (12%)	6 (10%)	3 (17%)	1.18 (0.22-6.44)
Verbal Memory	20 (20%)	12 (16%)	8 (32%)	2.39 (0.84-6.79
Visual Memory	10 (10%)	3 (4%)	7 (30%)	10.21 (2.38-43.85)
Psychomotor Speed	20 (11%)	11 (15%)	9 (41%)	2.63 (0.82-8.40)
Reaction Time	10 (11%)	6 (9%)	4 (18%)	2.22 (0.56-8.75)
Complex Attention	9 (11%)	6 (10%)	3 (15%)	1.62 (0.37-7.12)
Cognitive Flexibility	16 (19%)	11 (17%)	5 (25%)	1.58 (0.47-5.25)

## References

[1] Vanderlind WM, Rabinovitz BB, Miao IY (2021) A systematic review of neuropsychological and psychiatric sequalae of COVID-19: implications for treatment. Curr Opin Psychiatry 34(4):420–33. 10.1097/YCO.0000000000000713 34016818 PMC8183238

[2] Taquet M, Geddes JR, Husain M, Luciano S, Harrison PJ (2021) 6-month neurological and psychiatric outcomes in 236 379 survivors of COVID-19: a retrospective cohort study using electronic health records. Lancet Psychiatry 8(5):416–27. 10.1016/S2215-0366(21)00084-5 33836148 PMC8023694

[3] Graham EL, Clark JR, Orban ZS, (2021) Persistent neurologic symptoms and cognitive dysfunction in non-hospitalized Covid-19 “long haulers”. Ann Clin Transl Neur 8(5):1073–85. 10.1002/acn3.51350 PMC8108421 33755344

[4] Rogers JP, Watson CJ, Badenoch J, (2021) Neurology and neuropsychiatry of COVID-19: a systematic review and meta-analysis of the early literature reveals frequent CNS manifestations and key emerging narratives. J Neurol Neurosurg Psychiatry 92(9):932–41. 10.1136/jnnp-2021-326405 34083395

[5] Bowles KH, McDonald M, Barron Y, Kennedy E, O’Connor M, Mikkelsen M (2021) Surviving COVID-19 After Hospital Discharge: Symptom, Functional, and Adverse Outcomes of Home Health Recipients. Ann Intern Med 174(3):316–25. 10.7326/M20-5206 33226861 PMC7707212

[6] de Graaf MA, Antoni ML, Ter Kuile MM, (2021) Short-term outpatient follow-up of COVID-19 patients: A multidisciplinary approach. EClinicalMedicine 32:100731. 10.1016/j.eclinm.2021.100731 33532720 PMC7843037

[7] Garrigues E, Janvier P, Kherabi Y, (2020) Post-discharge persistent symptoms and health-related quality of life after hospitalization for COVID-19. J Infection 81(6):E4–E6. 10.1016/j.jinf.2020.08.029 PMC7445491 32853602

[8] Mannan A, Mehedi HMH, Chy N, (2021) A multi-centre, cross-sectional study on coronavirus disease 2019 in Bangladesh: clinical epidemiology and short-term outcomes in recovered individuals. New Microbes New Infect 40:100838. 10.1016/j.nmni.2021.100838 33520252 PMC7834423

[9] Almeria M, Cejudo JC, Sotoca J, Deus J, Krupinski J (2020) Cognitive profile following COVID-19 infection: Clinical predictors leading to neuropsychological impairment. Brain Behav Immun Health 9:100163. 10.1016/j.bbih.2020.100163 33111132 PMC7581383

[10] Liu YH, Wang YR, Wang QH, (2021) Post-infection cognitive impairments in a cohort of elderly patients with COVID-19. Mol Neurodegener 16(1):48. 10.1186/s13024-021-00469-w 34281568 PMC8287105

[11] Raman B, Cassar MP, Tunnicliffe EM, (2021) Medium-term effects of SARS-CoV-2 infection on multiple vital organs, exercise capacity, cognition, quality of life and mental health, post-hospital discharge. EClinicalMedicine 31:100683. 10.1016/j.eclinm.2020.100683 33490928 PMC7808914

[12] Soldati AB, Almeida C, Lima M, Araujo A, Araujo-Leite MA, Silva MTT (2021) Telephone Screening of Cognitive Status (TICS) in severe COVID-19 patients: Utility in the era of social isolation. eNeurologicalSci 22:100322. 10.1016/j.ensci.2021.100322 33495738 PMC7817447

[13] Tomasoni D, Bai F, Castoldi R, (2021) Anxiety and depression symptoms after virological clearance of COVID-19: A cross-sectional study in Milan, Italy. J Med Virol 93(2):1175–79. 10.1002/jmv.26459 32841387 PMC7461061

[14] Woo MS, Malsy J, Pottgen J, (2020) Frequent neurocognitive deficits after recovery from mild COVID-19. Brain Commun 2(2):fcaa205. 10.1093/braincomms/fcaa205 33376990 PMC7717144

[15] Becker JH, Lin JJ, Doernberg M, (2021) Assessment of Cognitive Function in Patients After COVID-19 Infection. JAMA Netw Open 4(10):e2130645. 10.1001/jamanetworkopen.2021.30645 34677597 PMC8536953

[16] Halalau A, Imam Z, Karabon P, (2021) External validation of a clinical risk score to predict hospital admission and in-hospital mortality in COVID-19 patients. Ann Med 53(1):78–86. 10.1080/07853890.2020.1828616 32997542 PMC7877986

[17] World Health Organization (2021). Clinical management of COVID-19: living guidance. https://www.who.int/publications/i/item/WHO-2019-nCoV-clinical-2021-1. Accessed 18 November 2021

[18] Gualtieri CT (2007) An Internet-based symptom questionnaire that is reliable, valid, and available to psychiatrists, neurologists, and psychologists. MedGenMed 2007 9(4):3 PMC2234274 18311353

[19] Gualtieri CT, Johnson LG (2006) Reliability and validity of a computerized neurocognitive test battery, CNS Vital Signs. Arch Clin Neuropsych 21(7):623–43. 10.1016/j.acn.2006.05.007 17014981

[20] CNS-Vital Signs (2020) CNS Vital Signs Interpretation Guide White Paper. https://www.cnsvs.com/WhitePapers/CNSVS-BriefInterpretationGuide.pdf. Accessed 18 November 2021

[21] Guilmette TJ, Sweet JJ, Hebben N, (2020) American Academy of Clinical Neuropsychology consensus conference statement on uniform labeling of performance test scores. Clin Neuropsychol 34(3):437–53. 10.1080/13854046.2020.1722244 32037942

[22] Benjamini Y, Hochberg Y (1995) Controlling the False Discovery Rate: A practical and powerful approach to multiple testing. Journal of the Royal Statistical Society B 57:289–300

[23] Cabral CMN, Miyamoto GC, Franco KFM, Bosmans JE (2021) Economic evaluations of educational, physical, and psychological treatments for fibromyalgia: a systematic review with meta-analysis. Pain 162(9):2331–45. 10.1097/j.pain.0000000000002233 33605655

[24] Wortman MSH, Lokkerbol J, van der Wouden JC, Visser B, van der Horst HE, Olde Hartman TC (2018) Cost-effectiveness of interventions for medically unexplained symptoms: A systematic review. PLoS One 13(10):e0205278. 10.1371/journal.pone.0205278 30321193 PMC6188754

[25] Dressing A, Bormann T, Blazhenets G, (2021) Neuropsychological profiles and cerebral glucose metabolism in neurocognitive Long COVID-syndrome. J Nucl Med. 10.2967/jnumed.121.262677 PMC9258569 34649946

[26] Mendez R, Balanza-Martinez V, Luperdi SC, (2021) Short-term neuropsychiatric outcomes and quality of life in COVID-19 survivors. J Intern Med. 10.1111/joim.13262 PMC8013333 33533521

[27] Zhou HT, Lu SJ, Chen JK, (2020) The landscape of cognitive function in recovered COVID-19 patients. J Psychiatr Res 129:98–102. 10.1016/j.jpsychires.2020.06.022 32912598 PMC7324344

[28] Brownlee NNM, Wilson FC, Curran DB, Lyttle N, McCann JP (2020) Neurocognitive outcomes in adults following cerebral hypoxia: A systematic literature review. NeuroRehabilitation 47(2):83–97. 10.3233/NRE-203135 32716324

[29] Pandharipande PP, Girard TD, Jackson JC, (2013) Long-term cognitive impairment after critical illness. N Engl J Med 369(14):1306–16. 10.1056/NEJMoa1301372 24088092 PMC3922401

[30] Thakur KT, Miller EH, Glendinning MD, (2021) COVID-19 neuropathology at Columbia University Irving Medical Center/New York Presbyterian Hospital. Brain 144(9):2696–2708. 10.1093/brain/awab148 33856027 PMC8083258

[31] Zhou Y, Xu J, Hou Y, (2021) Network medicine links SARS-CoV-2/COVID-19 infection to brain microvascular injury and neuroinflammation in dementia-like cognitive impairment. Alzheimers Res Ther 13(1):110. 10.1186/s13195-021-00850-3 34108016 PMC8189279

[32] Prudencio M, Erben Y, Marquez CP, (2021) Serum neurofilament light protein correlates with unfavorable clinical outcomes in hospitalized patients with COVID-19. Sci Transl Med 13(602). 10.1126/scitranslmed.abi7643 PMC8432951 34131052

[33] Magusali N, Graham AC, Piers TM, (2021) A genetic link between risk for Alzheimer’s disease and severe COVID-19 outcomes via the OAS1 gene. Brain. 10.1093/brain/awab337 PMC8500089 34619763

[34] Munshi MN (2017) Cognitive Dysfunction in Older Adults With Diabetes: What a Clinician Needs to Know. Diabetes Care 40(4):461–67. 10.2337/dc16-1229 28325796

[35] Teixeira MM, Passos VMA, Barreto SM, (2020) Association between diabetes and cognitive function at baseline in the Brazilian Longitudinal Study of Adult Health (ELSA-Brasil). Sci Rep 10: 1596. 10.1038/s41598-020-58332-9 32005901 PMC6994611

[36] Bruce BK, Allman ME, Rivera FA, (2020) Intensive Multicomponent Fibromyalgia Treatment: A Translational Study to Evaluate Effectiveness in Routine Care Delivery. J Clin Rheumatol. 10.1097/RHU.0000000000001555 32897994

[37] Cysique LA, Lojek E, Cheung TC, (2021) Assessment of Neurocognitive Functions, Olfaction, Taste, Mental, and Psychosocial Health in COVID-19 in Adults: Recommendations for Harmonization of Research and Implications for Clinical Practice. J Int Neuropsychol Soc 1–19. 10.1017/S1355617721000862 PMC8825876 34365990

