# Supplementary material for: Neurocognitive Screening in Patients Following SARS-CoV-2 Infection: Tools for Triage
Source: Res Sq. 2022 Feb 7:rs.3.rs-1127420. Preprint. [Version 1] doi: 10.21203/rs.3.rs-1127420/v1 (PMC8845509; doi:10.21203/rs.3.rs-1127420/v1)
Supplement: Supplement 1 [file NIHPPrs1127420v1-supplement-1.pdf]

Supplementary Table 1. Sociodemographic and clinical characteristics of patients lost to follow-up.

	**Completed outcome assessments (n=102)**	**Did not complete outcome assessments (n=47)**	***p-value***
**Age**, mean (SD)	52.55 (14.64)	48.62 (16.55)	0.15
**COVID-19 Severity Classification,** n (%)			0.007
Ambulatory	77 (76%)	44 (94%)	
Hospitalized	25 (24%)	3 (6%)	
**Sex**, n (%)			0.72
Males	42 (41%)	21 (45%)	
Females	60 (59%)	26 (55%)	
**Race,** n (%)			0.58
American Indian	0 (0 %)	0 (0%)	
Asian	5 (5%)	1 (2%)	
Native Hawaiian	0 (0%)	0 (0%)	
Black	9 (9%)	2 (4%)	
White	87 (85%)	43 (92%)	
More than one race	1 (1%)	1 (2%)	
**Ethnicity,** n (%)			0.46
Hispanic	5 (5%)	4 (9%)	
Non-Hispanic	97 (95%)	43 (91%)	

Supplementary Table 2. Objective Performance on neurocognitive test domains by group.

	**Overall (N=102)**	**Ambulatory (N=76)**	**Hospitalized (N= 26)**	***p-value***
**Group mean and variance by cognitive domain (age-adjusted based on normative reference group; standardized scale: mean=100, SD=15)**				
Neurocognitive Index, mean (SD)	95.59 (11.14)	95.93 (11.14)	94.41 (11.39)	0.623
Verbal Memory, mean (SD)	94.04 (18.27)	95.79 (17.31)	89.00 (20.32)	0.110
Visual Memory, mean (SD)	99.85 (14.36)	101.78 (12.62)	93.83 (17.80)	0.020*
Psychomotor Speed, mean (SD)	94.03 (15.58)	95.45 (14.31)	89.23 (18.87)	0.109
Reaction Time, mean (SD)	92.78 (11.65)	93.85 (11.63)	89.47 (11.32)	0.136
Complex Attention, mean (SD)	101.68 (13.32)	102.03 (13.09)	100.58 (14.30)	0.682
Cognitive Flexibility, mean (SD)	95.78 (15.48)	96.75 (15.18)	92.63 (16.41)	0.314
**Group proportions of patients showing impaired performance (<9th percentile) by cognitive domain**				
**Neurocognitive Index,** n (%)				0.453
Within normal limits	68 (88%)	53 (90%)	15 (83%)	
Impaired	9 (12%)	6 (10%)	3 (17%)	
**Verbal Memory,** n (%)				0.096
Within normal limits	78 (80%)	61 (84%)	17 (68%)	
Impaired	20 (20%)	12 (16%)	8 (32%)	
**Visual Memory,** n (%)				<0.001*
Within normal limits	86 (90%)	70 (96%)	16 (70%)	
Impaired	10 (10%)	3 (4%)	7 (30%)	
**Psychomotor Speed,** n (%)				0.008*
Within normal limits	76 (89%)	63 (85%)	13 (59%)	
Impaired	20 (11%)	11 (15%)	9 (41%)	
**Reaction Time,** n (%)				0.245
Within normal limits	78 (89%)	60 (91%)	18 (82%)	
Impaired	10 (11%)	6 (9%)	4 (18%)	
**Complex Attention,** n (%)				0.524
Within normal limits	72 (89%)	55 (90%)	17 (85%)	
Impaired	9 (11%)	6 (10%)	3 (15%)	
**Cognitive Flexibility,** n (%)				0.456
Within normal limits	67 (81%)	52 (83%)	15 (75%)	
Impaired	16 (19%)	11 (17%)	5 (25%)	
